# Effects of noninvasive brain stimulation on dual-task performance in different populations: A systematic review

**DOI:** 10.3389/fnins.2023.1157920

**Published:** 2023-04-11

**Authors:** Xiaoying Lin, Yanming Zhang, Xi Chen, Lifen Wen, Lian Duan, Lei Yang

**Affiliations:** ^1^Department of Rehabilitation Medicine, The Second People’s Hospital of Kunming, Yunnan Province, China; ^2^School of Rehabilitation, Kunming Medical University, Yunnan Province, China; ^3^Department of Rehabilitation Medicine, Xuanwu Hospital, Capital Medical University, Beijing, China; ^4^Department of Manipulation, Yuxi Hospital of Traditional Chinese Medicine, Yunnan Province, China

**Keywords:** noninvasive brain stimulation, transcranial direct current stimulation, transcranial magnetic stimulation, dual-task, systematic review

## Abstract

**Background:**

Increasing research has investigated the use of noninvasive brain stimulation (NIBS) on augmenting dual-task (DT) performance.

**Objective:**

To investigate the effects of NIBS on DT performance in different populations.

**Methods:**

Extensive electronic database search (from inception to November 20, 2022) was conducted in PubMed, Medline, Cochrane Library, Web of Science and CINAHL to identify randomized controlled trials (RCTs) that investigated the effects of NIBS on DT performance. Main outcomes were balance/mobility and cognitive function under both single-task (ST) and DT conditions.

**Results:**

Fifteen RCTs were included, involving two types of intervention techniques: transcranial direct current stimulation (tDCS) (twelve RCTs) and repetitive transcranial magnetic stimulation (rTMS) (three RCTs); and four different population groups: healthy young adults, older adults, Parkinson’s disease (PD), and stroke. For tDCS, under DT condition, significant improvement in speed was only observed in one PD and one stroke RCT, and stride time variability in one older adults RCT. Reduction in DTC in some gait parameters was demonstrated in one RCT. Only one RCT showed significant reduction in postural sway speed and area during standing under DT condition in young adults. For rTMS, significant improvements in fastest walking speed and time taken to Timed-up-and-go test under both ST and DT conditions were observed at follow-up in one PD RCT only. No significant effect on cognitive function in any RCT was observed.

**Conclusion:**

Both tDCS and rTMS showed promising effects in improving DT walking and balance performance in different populations, however, due to the large heterogeneity of included studies and insufficient data, any firm conclusion cannot be drawn at present.

## Introduction

Performing two tasks simultaneously (i.e., dual-task (DT) conditions) are common scenarios in many activities of daily living, for example, walking while talking, or while negotiating obstacles (Hillel et al., 2019). Therefore, how to improve the DT function is a topic of significance. Although a few systematic reviews have examined the efficacy of DT training on DT performance in different population (Delong and Wichmann, 2015; Yang et al., 2018; De Freitas et al., 2020), the optimal strategy of improving DT function in different population is yet to be developed.

Performing DT activities is the result of interplay of different structures of the central nervous system: the dorsolateral prefrontal cortex (DLPFC), supplementary motor area (SMA), primary motor cortex (M1), and cerebellum (Vitorio et al., 2017). As compared to walking alone, additional attention and cognitive resources are required in these challenging conditions, thus making the two tasks compete for the limited cognitive resources (Tombu and Jolicoeur, 2003), which will lead to DT “costs” (i.e., decrements) in motor and/or cognitive task performance (Lundin-Olsson et al., 1997; Yang et al., 2018). Among them, the DLPFC, a brain region that plays a critical role in executive functions, mainly mediates the cognitive process involved in dual-tasking (Weiss et al., 2015).

At present, there are two types of non-invasive brain stimulation (NIBS): transcranial magnetic stimulation (TMS) and transcranial direct current stimulation (tDCS), which uses electricity or magnetic flux to stimulate the intracranial neural tissues in a non-invasive way to regulate the excitability of central nerves and may induce lasting changes in neural plasticity, thus improving the function of subjects (Hara et al., 2021). Recent NIBS studies have shown their promising potential in the rehabilitation of neurological diseases. For example, it has been found that TMS was effective when combined with conventional training in improving depression, cognitive function, upper limb motor function, balance and gait after stroke (Zhang et al., 2017; Begemann et al., 2020; Behrangrad et al., 2021; Hara et al., 2021; Xie et al., 2021). tDCS has also demonstrated its effects on enhancing learning, working memory, executive planning, picture naming, and motor recovery in healthy young adults (Nitsche et al., 2003; Fregni et al., 2005; Dockery et al., 2009), as well as in the treatment of bipolar depression patients (D’Urso et al., 2023). Nevertheless, current studies that investigated the effects of NIBS on DT performance in different populations are scarce. Only several studies suggested that the NIBS may reduce the cost of performing a cognitive task when combined with a ambulation or postural control task in different populations (Wrightson et al., 2015; Zhou et al., 2015; Manor et al., 2018).

To sum up, since the NIBS has demonstrated facilitating motor and cognitive process separately in different populations, it was postulated that NIBS would be potentially useful in performing motor and cognitive tasks simultaneously, i.e., improving DT performance. However, whether using NIBS can address motor-cognitive interference in different populations is yet to be explored. Therefore, the aim of this systematic review was to investigate the effects of NIBS on DT performance in different populations.

## Methods

This systematic review was conducted according to the PRISMA guidelines (Page et al., 2021).

### Search strategy

Two independent investigators performed extensively searches using the following databases: PubMed, Medline, Cochrane Library, Web of Science and CINAHL. The literature search was performed using the keyword combination: [(NIBS) OR (TMS) OR (tDCS)] AND [(dual-task) OR (cognitive-motor)] AND [(walking) OR (gait) OR (balance)]. In addition, we also performed forward searches with Web of Science, and screened the reference list of each included publication so as not to miss any potential literature that met our criteria. The last search was conducted on December 20, 2022. Details of search strategy for the PubMed were provided in Appendix 1. Similar strategies were adapted to other databases.

### Eligibility criteria

The inclusion criteria were constructed as follows: (1) Both groups received the same intervention, except that the experimental group received TMS or tDCS intervention, while the control group received sham stimulation or no stimulation; (2) The outcomes involved the measures of motor or cognitive performance under DT condition; (3) The study design was randomized controlled RCT (RCT). The exclusion criteria were: (1) Case reports, non-experimental results, letters to the editor, conference reports, dissertations and reviews; (2) The full text of the publication was unavailable, despite contacted the authors.

### Assessment of methodological quality

The methodological quality of each included publication was assessed by using the Cochrane risk of bias tool (The Cochrane Collaboration), which is rated by the five dimensions: selection bias, performance bias, detection bias, attrition bias and reporting bias, with a full score of 12^1^. A study that does not meet the six criteria items or has a fatal defect is considered to have a high risk of bias. For example, if the drop-out rate is greater than 50%, it is considered as a fatal defect.

Two independent investigators (LXY, ZYM) jointly analyzed and determined the risk of bias for each publications, any disagreement between them was discussed and resolved with the principal investigator (YL).

### Data extraction and synthesis

Two independent investigators (LXY, ZYM) firstly screened the title and abstract of the searched publications. Then, the eligibility was further identified through full-text reading. If there was any disagreement, discussed and resolved with the third investigator (YL).

For the eligible publications, the first author extracted the general information about the study, e.g., participants’ characteristics, intervention protocols, and outcome measures. The primary outcomes extracted were walking (speed, step length, cadence, etc) and balance (center of pressure-related parameters) measures under both single - task (ST) and DT condition, as well as the corresponding DT-cost (DTC), while the secondary outcomes were cognitive performance under both ST and DT conditions, and other functions.

Due to the large heterogeneity of the included studies (different populations, different measures and small number of eligible studies), meta-analysis for each outcome was not performed. However, if the between-group comparison was significant, in order to facilitate the comparison across RCTs, we calculated the effect size (Hedges’ g) of gait, balance, cognition and other parameters under ST and DT conditions based on the original data given in the publications. For the follow-up results, we calculated the Hedges’ g between the follow-up value and the pre-intervention value, in order to check whether the treatment effect still exists at the follow-up.

## Result

### Article selection and methodology assessment

**Figure 1:**
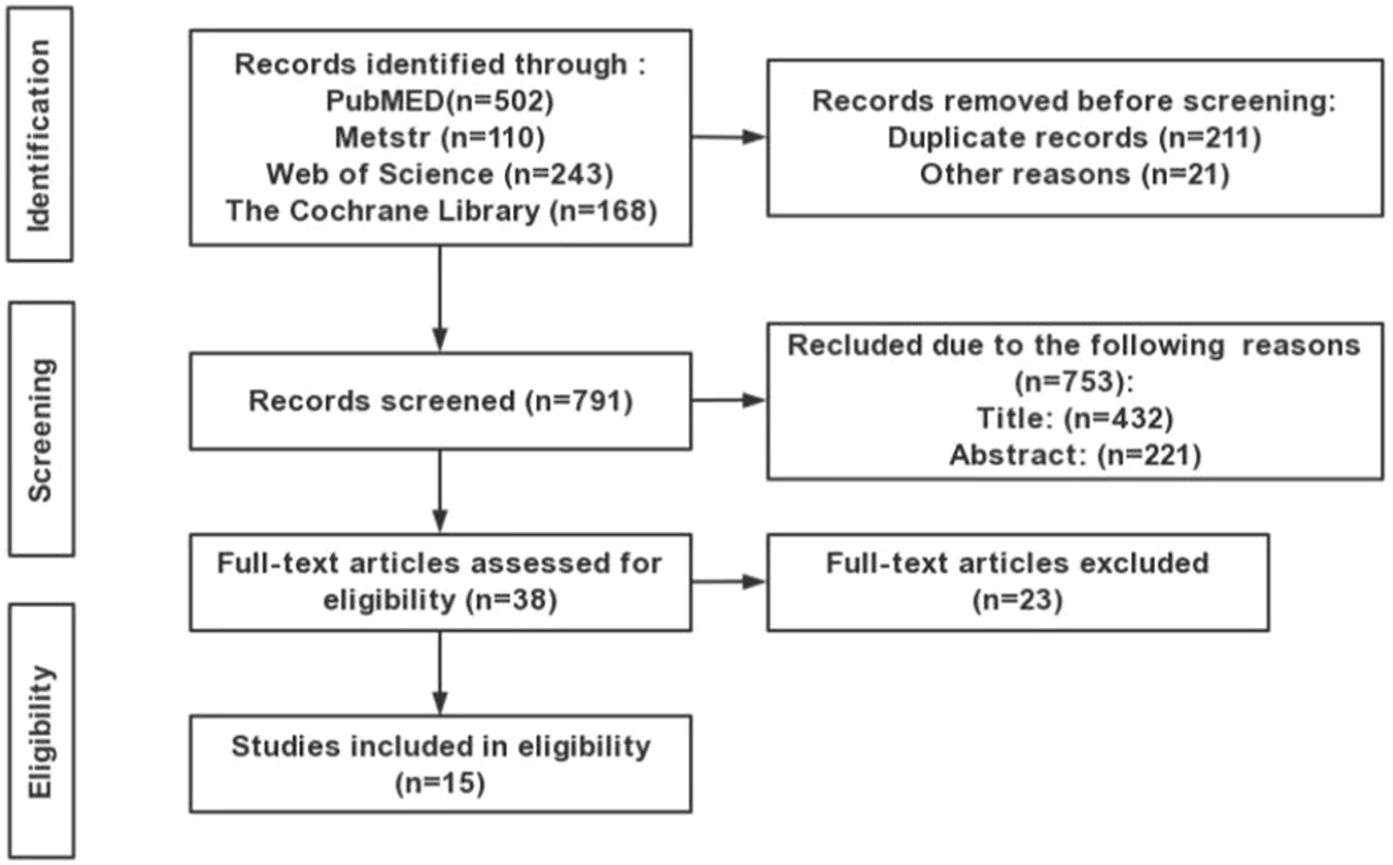
Flow chart.

The literature search process is shown in Figure 1, 1,023 publications were generated from the electronic search. After screening, 1,008 articles did not meet the inclusion criteria, thus were removed. Finally, 15 randomized controlled RCTs (RCTs) were identified in this review. The methodological quality of included RCTs was summarized in Table 1. The total risk of bias assessment score of the 15 included RCTs ranged from 7 to 10, indicating “low risks” (Table 1).

**Table 1:** Risk of bias.

Study	Randomization adequate	Concealed allocation	Blind subjects	Blind therapists	Blind assessors	Was the drop-out rate described and acceptable?	Were all randomized participants analyzed in the group to which they were allocated?	Free of selective outcome reporting?	Similarity of baseline characteristics	Cointerventions avoided or similar	Compliance acceptable	Timing of the outcome assessments similar	Total risk of bias score
rTMS trials (*N* = 3)													
Goh et al. (2020)	✓	–	–	?	–	✓	✓	✓	✓	?	✓	✓	7
Goh et al. (2019)	✓	–	–	?	?	✓	✓	✓	✓	?	✓	✓	7
Chung et al. (2020)	✓	✓	✓	?	✓	✓	✓	✓	✓	?	✓	✓	10
*tDCS trials*													
Zhou et al. (2014)	✓	✓	✓	?	✓	✓	✓	✓	✓	?	✓	✓	10
Wrightson et al. (2015)	✓	✓	✓	?	✓	✓	✓	✓	✓	?	✓	✓	10
Pineau et al. (2021)	✓	–	✓	?	✓	✓	✓	✓	✓	?	✓	✓	9
Zhou et al. (2015)	✓	✓	✓	?	✓	✓	✓	✓	✓	?	✓	✓	10
Manor et al. (2016)	✓	✓	✓	?	✓	✓	✓	✓	✓	?	✓	✓	10
Manor et al. (2018)	✓	✓	✓	?	✓	✓	✓	✓	✓	?	✓	✓	10
Schneider et al. (2021)	✓	✓	✓	?	✓	✓	✓	✓	✓	?	✓	✓	10
Schabrun et al. (2016)	✓	✓	✓	?	✓	✓	✓	✓	✓	?	✓	✓	10
Swank et al. (2016)	✓	✓	✓	?	✓	✓	✓	✓	✓	?	✓	✓	10
Mishra and Thrasher (2021)	✓	✓	✓	?	✓	✓	✓	✓	✓	?	✓	✓	10
Wong et al. (2022a,b)	✓	✓	✓	?	✓	✓	✓	✓	✓	?	✓	✓	10
Wong et al. (2022a,b)	✓	✓	✓	?	✓	✓	✓	✓	✓	?	✓	✓	10

✓ = yes; − = no; ? = unclear.

### Participants’ characteristics and intervention protocols

The demographics were summarized in Table 2, involving two types of intervention techniques: repetitive TMS (rTMS) (three RCTs) and tDCS (twelve RCTs); and four different population groups: healthy young adults (four RCTs, *N* = 73), older adults (four RCTs, *N* = 100), PD (five RCTs, *N* = 132), and stroke (two RCTs, *N* = 63). The average age of participants ranged from 21.1 (1) to 82 (4) years.

**Table 2:** Characteristics of participants and intervention protocols.

Study	Characteristics of participants			Intervention protocols			
	Population and sample size (ratio of female)	Age (y) [Mean (SD)]	Disease onset duration (y)	EG	CG	Intervention period	Additional therapy
rTMS (*N* = 3)							
Goh et al. (2019)	Young EG1: 9 (55.6%) EG2: 10 (60%)	29.3 (5.8)	--	Site: EG1: DLPFC; EG2: SMA Parameters: 90% RMT, 5 Hz, ITI of 30s, 1,200 pulses	Site: the left M1 Parameters: 90% RMT, 5 Hz, ITI of 30s, 1,200 pulses	1 session	NR
Goh et al. (2020)	Stroke 15 (33%)	57.7 (9.7)	22.8 (16.7)	Site: EG1: DLPFC; EG2: SMA Parameters: 90% RMT, 5 Hz, ITI of 30s, 1,200 pulses	Site: the left M1 Parameters: 90% RMT, 5 Hz, ITI of 30s, 1,200 pulses	1 session	NR
Chung et al. (2020)	PD EG1: 17 (41%) EG2: 17 (47%) CG: 16 (56%)	EG1: 62.7 (6.8) EG2: 62.1 (5.7) CG: 62.1 (5.7)	EG: 5.2 (3.4) EG2: 7.5 (4.9) CG: 6.9 (3.3)	Site: the leg area of bilateral M1 Parameters: EG1: 25 Hz; EG2: 1 Hz; 80% RMT, ITI of 50s, 1,200 pulses	Site: the leg area of bilateral M1 Parameters: sham	4 days/week, for 3 weeks (12 sessions)	30 min of treadmill training.
tDCS (*N* = 12)							
Zhou et al. (2014)	Young 20 (50%)	22 (2)	--	Site: Anode: L-DLPFC; Cathoda: R- supraorbital region Parameters: 1.1 ± 0.3 mA, 20 min	Site: Anode: L-DLPFC; Cathoda: R- supraorbital region Parameters: 0 mA, 20 min	1 session	NR
Wrightson et al. (2015)	Young 10 (NR)	23 (3.2)	--	Site: EG1: Anode: PFC; EG2: Cathoda: PFC Parameters: 1.5 mA, 15 min	Site: PFC Parameters: 0 mA, 0.5 min	1 session	NR
Pineau et al. (2021)	Young EG: 12 (25%) CG: 12 (25%)	EG: 21.6 (1.6) CG: 21.1 (1.0)	--	Site: Anode:L-DLPFC; Cathoda:R- supraorbital region Parameters: 2 mA/ 30 min	Site: Anode:L-DLPFC; Cathoda:R- supraorbital region Parameters: 2 mA/ 1 min	1 session	NR
Zhou et al. (2015)	Older 20 (45%)	63 (3.6)	--	Site: Anode: L-DLPFC; Cathoda: R- supraorbital region Parameters: 1.4 ± 0.4 mA/ 20 min	Site: Anode:L-DLPFC; Cathoda:R- supraorbital region Parameters: 2 mA/ 1 min	1 session	NR
Manor et al. (2016)	Older 37 (67.6%)	61 (5.0)	--	Site: Anode:L-DLPFC; Cathoda:R- supraorbital region Parameters: 1.4 ± 0.4 mA/ 20 min	Site: Anode:L-DLPFC; Cathoda:R- supraorbital region Parameters: 2 mA/ 1 min	1 session	NR
Manor et al. (2018)	Older EG: 9 (55.6%) CG: 9 (55.6%)	EG: 82 (4.0) CG: 79 (4.0)	--	Site: Anode:L-DLPFC; Cathoda:R- supraorbital region Parameters: 1.9 ± 0.3 mA/ 20 min	Site: Anode:L-DLPFC; Cathoda:R- supraorbital region Parameters: 2.0 ± 0.1 mA/ 1 min	5 days/week, for 2 weeks (10 sessions)	NR
Schneider et al. (2021)	Older 25 (80%)	73.9 (5.2)	--	Site: M1 + LDLPFC Parameters: 20 min	Site: M1 + LDLPFC Parameters: sham	1 session	EG: tDCS+walking; CG: sham+walking
Schabrun et al. (2016)	PD EG: 8 (NR) CG: 8 (NR)	EG: 72 (4.9) CG: 63 (11.0)	EG: 6.9 (4.4) CG: 4.6 (3.9)	Site: Anode:L-M1; Cathoda:R- supraorbital region Parameters: 2 mA/ 20 min	Site: Anode:L-M1; Cathoda:R- supraorbital region Parameters: 2 mA/ 0 min	3 days/week, for 3 weeks (9 sessions)	20 min of gait training
Swank et al. (2016)	PD 10 (NR)	68.7 (10.2)	7.9 (7.1)	Site: Anode:L-DLPFC; Cathoda:R- supraorbital region Parameters: 2 mA/ 20 min	Site: Anode: L-DLPFC; Cathoda: R- supraorbital region Parameters: 1 mA/ 0.5 min	1 session	NR
Mishra and Thrasher (2021)	PD 20 (NR)	67.8(8.3)	4.8 (3.8)	Site: Anode:L-DLPFC; Cathoda:R- supraorbital region Parameters: 2 mA/ 30 min	Site: Anode: L-DLPFC; Cathoda: R- supraorbital region Parameters: 2 mA/ 1 min	1 session	NR
Wong et al. (2022a,b)	PD EG1: 9 EG2: 9 EG3: 9 CG: 9	EG1: 54.20 (4.1) EG2: 50.09 (2.4) EG3: 61.30 (7.9) CG: 58.30 (8.0)	EG1: 7.8 (5.7) EG2: 6.2 (3.3) EG3: 4.1 (3.3) CG: 8.3 (0.12)	Site: Anode: EG1: L- M1; EG2: L-DLPFC; EG3: L-Cerebellum; Cathode: R- supraorbital region Parameters: 2 mA/ 20 min	Site: Anode: L- M1; Cathode: Contralateral supraorbital ridge Parameters: 2 mA/ 1 min	1 session	30 min of gait training
Wong et al. (2022a,b)	Stroke EG1: 12 EG2: 12 EG3: 12 CG: 12	EG1:54.3 (16.1) EG2: 53.3 (19.0) EG3: 59.2 (12.7) CG: 55.2 (14.0)	EG1:59.9 (57.3) EG2: 63.0 (40.8) EG3: 57.8 (71.3) CG: 57.4 (58.2)	Site: EG1: Anode: ipsilesional M1; Cathode: contralateral supraorbital ridge; EG2: Anode: ipsilesional M1; Cathoda:contralesional M1; EG3: Anode: contralateral supraorbital ridge; Cathode: contralesional M1 Parameters: 2 mA/ 20 min	Site: Anode: ipsilesional M1; Cathode: contralateral supraorbital ridge Parameters: 2 mA/ 1 min	1 session	NR

CG: control group; EG: experimental group; L: left; NR: not reported; PD: Parkinson’s Disease; R: right.

#### rTMS protocols

Three RCTs investigated the effects of rTMS in healthy young adults, individuals with stroke and individuals with PD respectively, with different stimulation protocols. Goh et al. (2019, 2020) applied one single-session 5 Hz rTMS to healthy young adults and individuals with stroke. The stimulation targets of the experimental group were in the left DLPFC or SMA, while the control group was in the M1 (Goh et al., 2019, 2020). Chung et al. (2020) set the target over the leg area of bilateral M1 with three different groups (1 Hz, 25 Hz and sham stimulation) for 12 sessions.

#### tDCS protocols

The effects of tDCS were examined in 12 RCTs, involving four different populations, namely, healthy young adults, older adults, individuals with PD, and individuals with stroke. The anode was placed over the left DLPFC, left-M1, M1-LDLPFC, left cerebellum, or PFC, while the cathode were generally on the right supraorbital cortex (Zhou et al., 2014, 2015; Wrightson et al., 2015; Manor et al., 2016, 2018; Schabrun et al., 2016; Swank et al., 2016; Mishra and Thrasher, 2021; Pineau et al., 2021; Schneider et al., 2021; Wong et al., 2022a,b). Participants in most studies received only one session stimulation (Zhou et al., 2014, 2015; Wrightson et al., 2015; Manor et al., 2016; Swank et al., 2016; Mishra and Thrasher, 2021; Pineau et al., 2021; Schneider et al., 2021; Wong et al., 2022a,b). The intensities and duration of stimulation ranged from 1.1 to 2 mA, and 15 to 30 min, respectively.

### Effects of rTMS on walking ability

#### Healthy young adults and individuals with stroke

No significant improvement in speed was observed in healthy young adults and individual with stroke under either ST or DT condition (Goh et al., 2019, 2020; Table 3).

**Table 3:** NIBS: Effects on mobility outcomes.

Outcomes	tDCS (*N* = 10)		TMS (N = 3)	
	Under ST condition	Under DT condition	Under ST condition	Under DT condition
Walking function				
Healthy young adults (*N* = 3)	Speed^a^ (Pineau et al., 2021), Stride time variability (Wrightson et al., 2015): (NS)	Speed^a^ (Zhou et al., 2014), DTC in speed (Zhou et al., 2014), DTC in stride time variability (Wrightson et al., 2015): (NS)	Speed^a^ (Goh et al., 2019): (NS)	Speed^a^ (Goh et al., 2019): (NS)
Older adults (*N* = 3)	Speed (Manor et al., 2016, 2018), TUG (Manor et al., 2018), 4-m walking time (Manor et al., 2018), Stride time (Manor et al., 2018), Stride time variability and 2-week-FU (Manor et al., 2018): (NS)	Speed (Manor et al., 2016, 2018), Stride time (Manor et al., 2018), and their corresponding DTC: (NS) DTC in Stride time variability (Manor et al., 2018) and Double support time (Schneider et al., 2021): (NS) Stride time variability (Manor et al., 2018): −0.61*; DTC in Stride time (Schneider et al., 2021): 1.14*; Stride time variability (Schneider et al., 2021): 4.04*; Swing time variability (Schneider et al., 2021): 0.87*; Step regularity (Schneider et al., 2021): 0.46*		
Stroke (*N* = 2)	Step time (Wong et al., 2022a,b), Step length (Wong et al., 2022a,b), DTC in Speed (Wong et al., 2022a,b): (NS) Speed (Wong et al., 2022a,b): 0.37* Cadence (Wong et al., 2022a,b): 0.5*	Cadence (Wong et al., 2022a,b), Step time: (NS) (Wong et al., 2022a,b), Step length (Wong et al., 2022a,b), DTC in Speed (Wong et al., 2022a,b): (NS) Speed (Wong et al., 2022a,b): 0.27 ~ 0.34*	Speed^a^ (Goh et al., 2020): (NS)	Speed^a^ (Goh et al., 2020): (NS)
Parkinson’s disease (*N* = 4)	Speed (Schabrun et al., 2016; Mishra and Thrasher, 2021; Wong et al., 2022a,b), TUG (Schabrun et al., 2016; Swank et al., 2016; Wong et al., 2022a,b), Cadence (Schabrun et al., 2016; Wong et al., 2022a,b), Stride time (Wong et al., 2022a,b), Step length (Schabrun et al., 2016; Wong et al., 2022a,b), Double support time and 12-week FU(Schabrun et al., 2016): (NS)	Speed (Schabrun et al., 2016; Mishra and Thrasher, 2021), DTC in speed (Mishra and Thrasher, 2021; Wong et al., 2022a,b), TUG^a^ (Schabrun et al., 2016; Swank et al., 2016), Cadence: (NS) (Schabrun et al., 2016; Wong et al. 2022a,b), Stride time (Wong et al., 2022a,b), Step length (Schabrun et al., 2016; Wong et al., 2022a,b), Double support time and 12-week FU (Schabrun et al., 2016): (NS) Speed (Zhou et al., 2014): 0.65*	Fastest walking speed and 1-month FU (Chung et al., 2020): (NS) month FU (Chung et al., 2020): 0.51* TUG and 1-month FU (Chung et al., 2020): (NS) 3-month FU (Chung et al., 2020): −0.64*	TUG (Chung et al., 2020): (NS) 1-month FU (Chung et al., 2020): −0.67* 3-month FU (Chung et al., 2020): −0.59*
Balance function (laboratory-based measures)				
Healthy young adults (*N* = 3)	Postural sway speed and area^a^ (Zhou et al., 2014), Range AP and ML (Eyes open/closed) (Pineau et al., 2021), Mean velocity (Eyes open/closed) (Pineau et al., 2021): (NS)	ML/AP trunk RoM and their corresponding DTC (Wrightson et al., 2015; Pineau et al., 2021), DTC in postural sway speed and area^a^ (Zhou et al., 2014), Mean velocity (open/closed): (NS) (Pineau et al., 2021) Postural sway area and speed^a^* (Zhou et al., 2014)		
Older adults (*N* = 2)	COP complexity index^a^ (Zhou et al., 2015), Standing postural sway area, speed and 2-week-FU (Manor et al., 2016, 2018): (NS)	COP complexity index and its DTC^a^ (Zhou et al., 2015), Standing postural sway area, speed and their corresponding DTC (Manor et al., 2018; Mishra and Thrasher, 2021): (NS)		

a : Original data not reported.

* : Significant improvement in experimental group compared with control group, values are standardized effect sizes for significant results (Hedges’ g: 0.2 = small, 0.5 = medium, 0.8 = large).

AP: anterior–posterior; DTC: dual-task costs; ML: medio-lateral; NS: not significant; RoM: range of motion; TUG: timed up and go test.

#### Individuals with PD

Compared with the control group, significant improvements in fastest walking speed and time taken to TUG under both ST and DT conditions were only observed at follow-up in one RCT (Chung et al., 2020; Table 3).

### Effects of tDCS on walking ability

#### Healthy young adults

No significant improvements in speed or stride time variability under either ST or DT condition, DTC in speed, or in stride time variability was observed (Zhou et al., 2014; Wrightson et al., 2015; Table 3).

#### Older adults

Except the stride time variability under DT condition, none of gait parameters (speed, TUG, stride time) showed better improvement under ST or DT condition (Manor et al., 2018). On the contrary, some DTC measures, such as DTC in stride time, stride time variability, swing time variability, and step regularity demonstrated significant better improvement (Schneider et al., 2021; Table 3).

#### Individuals with PD

Except significant improvement in speed under DT condition was reported in one RCT only (Wong et al., 2022a,b), no significant improvement in any gait parameter was identified in other RCTs (Schabrun et al., 2016; Swank et al., 2016; Mishra and Thrasher, 2021; Table 3).

#### Individuals with stroke

Only one RCT demonstrated the significant improvement in speed under both ST and DT condition, and cadence in ST condition (Wong et al., 2022a,b; Table 3).

### Effects of tDCS on balance function

In five RCTs that investigated the balance function in healthy young adults and older adults, only one RCT showed significant reduction in postural sway speed and area during standing under DT condition (Zhou et al., 2014; Table 3).

### Effects of NIBS on cognitive function

No significant effect on cognitive function was observed in healthy young adults or older adults (Goh et al., 2019, 2020; Table 3).

### Effects of NIBS on other functions

#### Individuals with PD

Only one RCT reported that the rTMS could significantly improve the score of Movement Disorders Society-Unified Parkinson’s Disease Rating Scale part III (MDS-UPDRS III) at post-intervention and one-month follow-up, cortical silent period (CSP) at post-intervention, and short-interval intracortical inhibition (SICI) at one-month follow-up (Chung et al., 2020). No significant improvement in quality of life scores-39 was identified in the tDCS RCT (Swank et al., 2016). Only one trail demonstrated the tDCS could significantly lengthen CSP in DLPFC group (Wong et al., 2022a,b; Table 4).

**Table 4:** NIBS: Effects on cognition and other outcomes.

Outcomes	tDCS (*N* = 10)		TMS (*N* = 3)	
	Under ST condition	Under DT condition	Under ST condition	Under DT condition
Cognitive function				
Healthy young adults (*N* = 3)	Serial subtraction^a^ (Pineau et al., 2021), Error ratio: (NS) (Wrightson et al., 2015)	Serial subtraction^a^ (Pineau et al., 2021), DTC in Error ratio: (NS) (Wrightson et al., 2015)	Serial subtraction^a^ (Goh et al., 2020): (NS)	Serial subtraction^a^ (Goh et al., 2020): (NS)
Older adults (*N* = 4)	MoCA (Manor et al., 2018), TMT (Part B − Part A) (Manor et al., 2018), Stroop^a^ (Manor et al., 2016), Serial subtraction^a^ (Manor et al., 2016): (NS)	Serial subtraction error rate (Zhou et al., 2015), DTC in Serial subtraction^a^ (Mishra and Thrasher, 2021): (NS)		
Stroke (*N* = 1)			Serial subtraction^a^ (https://training.cochrane.org/handbook/current, see footnote 1): (NS)	Serial subtraction^a^ (https://training.cochrane.org/handbook/current, see footnote 1): (NS)
Parkinson’s disease (*N* = 3)	Words generated^a^ (Wong et al., 2022a,b): (NS)	Words generated (Swank et al., 2016), DTC in Words generated (Wong et al., 2022a,b), Serial subtraction (Swank et al., 2016; Schneider et al., 2021), DTC in Serial subtraction^a^ (Schneider et al., 2021): (NS)		
Other functions				
Parkinson’s disease (*N* = 3)	PDQ-39^a^ (Swank et al., 2016), Resting motor threshold (Zhou et al., 2014), Motor evoked potentials (Zhou et al., 2014): (NS) CSP (Zhou et al., 2014): 0.19*		Slope of RC (Goh et al., 2019), SICI (Goh et al., 2019): (NS) CSP: 0.55* (Goh et al., 2019) MDS-UPDRS III (Goh et al., 2019):-0.62* ~ −0.32* 1-mon follow-up (Goh et al., 2019): −0.51* SICI: 1-mon FU (Goh et al., 2019): −0.33* 3-mon FU (Goh et al., 2019): (NS)	
Stroke (*n* = 1)	Resting motor threshold (Schabrun et al., 2016), SICI (Schabrun et al., 2016): (NS) CSP (Schabrun et al., 2016): 0.39 ~ 0.48*			

a : Original data not reported.

* : Significant improvement in experimental group compared with control group; values are standardized effect sizes for significant results (Hedges’ g: 0.2 = small, 0.5 = medium, 0.8 = large).

CSP: cortical silent period; DTC:Dual-task costs; MDS-UPDRS III: Movement Disorders Society–Unifified Parkinson’s Disease Rating Scale part III; MoCA: Montreal Cognitive Assessment; NS: not significant; PDQ-39: quality of life scores-39; RC: recruitment curve; SICI: short-interval intracortical inhibition; TMT (Part B − Part A): Trail Making Test Part B minus Part A.

#### Individuals with stroke

Significant improvement of CSP was demonstrated by one tDCS RCT only (Wong et al., 2022a,b; Table 4).

## Discussion

### Findings of this review

A total of 15 RCTs were included in this review, comparing the effects of NIBS with sham-stimulation in different populations. Significant improvements in DT walking (speed, time taken to TUG, cadence) and balance (postural sway speed and area) performance were only observed in 3 (Chung et al., 2020; Wong et al., 2022a,b) and 1 (Zhou et al., 2014) RCTs, respectively. Similarly, reduction in DTC in some gait parameters (stride time, stride time variability, swing time variability, step regularity) was demonstrated in one RCT only (Schneider et al., 2021). In addition, due to the limited number and large heterogeneity of included RCTs, there was no evidence to suggest that NIBS was superior to sham-stimulation in improving DT walking and balance function.

### rTMS effects on mobility function

Chung et al. showed that rTMS (25 Hz, 1 Hz, or sham) applying to the leg area of bilateral M1 in individuals with PD followed by treadmill training could significantly improve the time taken to TUG and fastest walking speed under both ST and DT conditions, and MDS-UPDRS III scores at post-intervention and follow-up. This was similar to the results of Yang et al. (2018) study. The meta-analysis by Yang et al. (2018) investigated the optimal therapeutic effects of rTMS parameters on mobility dysfunction and provided evidence supporting that rTMS could be effective in improving mobility function in individuals with PD. Chung et al. (2020) and Yang et al. (2018) both concluded that high-frequency (25 Hz) rTMS could significantly improve cortical excitability in individuals with PD, this behavioral changes could be associated with increased cortical excitability. On one hand, rTMS can improve the neurological plasticity through the regulation of central nervous system (King and Tang, 2022); on the other hand, rTMS-primed treadmill training could strengthen synaptic connections in M1, which participates in the processing and storage of new information for motor consolidation, thereafter, leading to more stable and longer duration effect.

By contrast, in Goh et al. study, no significant improvement was identified in either healthy young adults or stroke under either ST or DT condition. This could be attributed to the only one-single session of stimulation was applied in their RCT, making no stimulation effect accumulated.

### tDCS effects on mobility function

Significant improvements in gait parameters were demonstrated by only one RCT, respectively, in stroke (ST condition: speed, cadence; DT condition: speed) (Wong et al., 2022a,b) and PD (DT condition: speed) (Wong et al., 2022a,b). Only one RCT reported the significant findings in DT postural sway speed and area (Zhou et al., 2014). Therefore, there is insufficient data on the effect of tDCS on mobility in the four studied populations at present. Future research should further investigate the effects of tDCS on DT mobility function in different population with larger sample size and longer intervention period.

The DTC in gait parameters, such as, stride time variability, swing time variability and step regularity, were significantly reduced (Schneider et al., 2021) in healthy young adults and older adults. Theoretically, under DT condition, cognitive resources would be divided, leading one or both tasks deteriorated (Sigman and Dehaene, 2006). However, the DT interference in walking was improved after tDCS intervention, this phenomenon could be mainly due to the activation of DLPFC by tDCS, which promotes the speed of task processing in the brain (Filmer et al., 2013), i.e., improving the executive function, thereby reducing the DTC. The assumption was supported by the fact that the stimulation site of the 7 included RCTs in this review was all on the PFC (DLPFC) (Healthy young adults: 3 studies; Older adults: 4 studies). Previous literature suggested that the DLPFC, particularly the left DLPFC, plays a critical role in regulating the execution of mobility-cognitive task, possibly due to its role in executive function (Beurskens et al., 2014; Liu et al., 2018). Alternatively, DLPFC is closely related to DT function. To sum up, tDCS applied to DLPFC seems to be able to decrease the DTC in gait parameters of healthy young adults and older adults.

Although the improvements in walking, balance, or cognition were generally not significant, many studies in recent years have shown that there was a tendency for NIBS to improve the modulating cortical efficiency in healthy young adults, older adults, individuals with stroke and individuals with PD (Cosentino et al., 2017; Ghosh et al., 2019; Bai et al., 2022). Theoretically, the behavioral or functional changes would occur after the plasticity changes. Since most RCTs in our review applied only one single-session stimulation (Zhou et al., 2014, 2015; Wrightson et al., 2015; Manor et al., 2016; Swank et al., 2016; Mishra and Thrasher, 2021; Pineau et al., 2021; Schneider et al., 2021; Wong et al., 2022a,b) or short intervention period (2–3 weeks) (Schabrun et al., 2016; Manor et al., 2018), the plasticity changes could have not occurred. In addition, the basal ganglia, central to movement disorders pathophysiology, could not be reached directly by tDCS or rTMS, but stimulation of appropriate cortical areas may affect activity in these circuits and may produce clinical benefit (Delong and Wichmann, 2015; Latorre et al., 2019).

### NIBS effects on cognitive function

Overall, the effect of NIBS on cognitive function was not significant under either ST or DT condition in any population studied. This is quite different from the results of previous systematic review and meta-analysis, despite the populations were different. In their review, tDCS could significantly improve attention/vigilance in different brain disorders (schizophrenia, depression, dementia, PD, MS, stroke, and TBI) (Begemann et al., 2020), and both tDCS and rTMS shows promising positive effects in attention, memory, and working memory for post-stroke patients with deficits in cognitive function (Hara et al., 2021). The inconsistency in findings may be mainly attributed to that only one single-session intervention (short period) adopted by most RCTs (Zhou et al., 2014, 2015; Wrightson et al., 2015; Manor et al., 2016; Swank et al., 2016; Goh et al., 2019, 2020; Mishra and Thrasher, 2021; Schneider et al., 2021), leading to no intervention effect could be accumulated. However, since there is lack of data, the effects of NIBS on DT cognitive function need to be further explored.

### Comparison of tDCS and rTMS effects on DT performance

Although tDCS showed significant improvement in DT walking speed in stroke (Wong et al., 2022a,b) and PD (Wong et al., 2022a,b) respectively in one RCT, while rTMS did not show any significant changes after intervention, the treatment effects of tDCS and rTMS were not comparable in different populations, due to the limited number of studies included in this review, different stimulation target, and the different intervention protocol adopted in each trail.

### Limitations

#### Limitations of studies reviewed

Small sample size (9 ~ 20) (Zhou et al., 2014, 2015; Wrightson et al., 2015; Schabrun et al., 2016; Swank et al., 2016; Manor et al., 2018; Goh et al., 2019, 2020; Mishra and Thrasher, 2021) and short intervention period (only one-single session) (Zhou et al., 2014, 2015; Wrightson et al., 2015; Manor et al., 2016; Swank et al., 2016; Goh et al., 2019, 2020; Mishra and Thrasher, 2021; Pineau et al., 2021; Schneider et al., 2021; Wong et al., 2022a,b) were adopted by most RCTs, making the results should be interpreted with caution.

#### Limitations of this systematic review

There are several limitations in our review. The publications were only screened in the English databases and may ignore potential publications in other languages. In addition, the meta-analysis was not performed due to the heterogeneity in stimulation parameters and populations studied of the included publications.

## Conclusion

Both tDCS and rTMS showed promising effects in improving DT walking and balance performance in different populations, however, due to the large heterogeneity of included studies and insufficient data, any firm conclusion cannot be drawn at present. More well-designed studies with longer intervention period and larger sample size are needed.

## Data availability statement

The raw data supporting the conclusions of this article will be made available by the authors, without undue reservation.

## Author contributions

All authors listed have made a substantial, direct, and intellectual contribution to the work and approved it for publication. XL, YZ, LD, and LY provided concept/idea/research design. XL and LY provided writing. XL, XC, and LW. provided data collection and quality assessment of the study. LW and LD provided data analysis. LY provided project management. All authors provided consultation (including review of manuscript before submission).

## Funding

This study was supported by the funding of Kunming Health Science and Technology Talent Training Project, Training Plan for Medical Science and Technology Descipline Leaders, No. 2022-SW (Leaders)-27.

## Conflict of interest

The authors declare that the research was conducted in the absence of any commercial or financial relationships that could be construed as a potential conflict of interest.

## Publisher’s note

All claims expressed in this article are solely those of the authors and do not necessarily represent those of their affiliated organizations, or those of the publisher, the editors and the reviewers. Any product that may be evaluated in this article, or claim that may be made by its manufacturer, is not guaranteed or endorsed by the publisher.

## Footnotes

^1^ https://training.cochrane.org/handbook/current
