# Supplementary material for: Effects of noninvasive brain stimulation on dual-task performance in different populations: A systematic review
Source: Front Neurosci. 2023 Apr 11;17:1157920. doi: 10.3389/fnins.2023.1157920 (PMC10128879; doi:10.3389/fnins.2023.1157920)
Supplement: Supplementary file 1 [file Table_1.docx]

Appendix 1. Search strategy

#1 NIBS

#2 noninvasive brain stimulation

#3 transcranial magnetic stimulation

#4 TMS

#5 transcranial direct current stimulation

#6 tDCS

#7 neuromodulation

#8 #2 OR #3 OR #4 OR #5 OR #6 OR #7

#9 dual-task

#10 dual-task

#11 cognitive-motor

#12 divided attention

#13 attention-demanding

#14 #9 OR #10 OR #11 OR #12 OR #13

#15 walking

#16 gait

#17 balance

#18 mobility

#19 #15 OR #16 OR #17 OR #18

#20 #8 AND #14 AND #19

#21 Limit to human English
